# Integrative variants, haplotypes and diplotypes of the CAPN3 and FRMD5 genes and several environmental exposures associate with serum lipid variables

**DOI:** 10.1038/srep45119

**Published:** 2017-03-23

**Authors:** Tao Guo, Rui-Xing Yin, Ling Pan, Shuo Yang, Liu Miao, Feng Huang

**Affiliations:** 1 Department of Cardiology, Institute of Cardiovascular Diseases, the First Affiliated Hospital, Guangxi Medical University, Nanning 530021, Guangxi, People’s Republic of China

## Abstract

To determine whether the integrative variants, haplotypes and diplotypes of the calpain 3 (*CAPN3*) and the FERM domain containing 5 genes (*FRMD5*) and several environmental exposures are associated with an implication in lipid homeostasis, which are associated with cardiovascular risk. Genotyping of the *CAPN3* rs4344713 and *FRMD5* rs524908 was performed by Sanger sequencing in 1,640 subjects (Jing, 819 and Han, 821). Multivariate analyses of covariance models that adjusted by age, gender, body mass index (BMI), blood pressure and lifestyle (smoking and drinking), were constructed using variants, haplotypes and diplotypes of the *CAPN3* rs4344713 and *FRMD5* rs524908 as predictors and changes in lipid variables. Significant associations with low-density lipoprotein cholesterol and apolipoprotein (Apo) B were found. Linkage disequilibrium with each other showed the haplotype-phenotype associations with triglyceride and ApoA1. This study also suggested pleiotropic associations of the *CAPN3*-*FRMD5* diplotypes with lipid variables. As potential confounders, diastolic blood pressure (DBP) and BMI were significantly associated with lipid variables. We conclude that integrative variants, haplotypes and diplotypes of the *CAPN3* rs4344713 and *FRMD5* rs524908, as well as DBP and BMI are associated with serum lipid variables in the Jing and Han populations.

The recently revised guidelines from the European Society of Cardiology/European Atherosclerosis Society (ESC/EAS) reflect an awareness that human genetic and cardiometabolic heterogeneity could be used to adjust population-based guidelines so that they are optimal for individuals^1^. Likewise, the summary of the 2016 conference preventive cardiovascular disease (CVD) sponsored by the ESC/EAS in Rome emphasizes that we must identify the roles of candidate genes that influence individual susceptibility to incident CVD^2^. Alone these lines, several loci have already been investigated for potential interaction between gene and lipid variable^3^. Two of these are the calpain 3 gene (*CAPN3*; OMIM 114240; 40-kb, 15q15.1-q21.1)^4^ and the FERM domain containing 5 gene (*FRMD5*; OMIM 616309; 15q15.3)^5^ that mapped to the long arm of chromosome 15 closely (Fig. 1). Their gene products play a central role in lipid metabolism^6^,^7^ and CVD risk^8^,^9^. Many genome-wide association studies (GWASs) and target single nucleotide polymorphism (SNP) studies have found significant polymorphisms of these two genes in different ethnic groups. The most frequent mutations, rs2412710 for *CAPN3* and rs2929282 for *FRMD5* are associated with blood lipid concentration in European Caucasian individuals^10^,^11^. Our aim was to clarify the association of *CAPN3* and *FRMD5* with serum lipid variables in the Chinese subjects. In this study, after selection of tagging SNPs by Haploview (Broad Institute of MIT and Harvard, Cambridge, MA, USA, version 4.2), we validated the two significant SNPs, *CAPN3* rs4344713 and *FRMD5* rs524908, in Han Chinese in Beijing, China chosen from the 1000 Genome Project Database.

The *CAPN3* contains 24 exons encoding for the 94-kDa Na^+^/Ca^2+^-dependent cysteine protease calpain-3^12^,^13^, which encodes several key mediators of lipid metabolism and oxidative phosphorylation^14^. Likewise, using a multivariate approach, a previous study reported a combination of SNPs, included SNPs in *FRMD5* that explains a significant part of the variability in the postprandial chylomicron and triglyceride (TG) response^15^. To date, GWASs have identified hundreds of genetic clues to cardiometabolic disease. However, they are much more likely to be near a gene area closely that causes abnormal cardiometabolic traits, than a similarly sized random set of loci^16^. The *CAPN3* rs4344713 and *FRMD5* rs524908 SNPs are located so close together within a neighbor region (Fig. 1) that the linkage disequilibrium (LD) between them cannot be neglected. It was previously reported that an association analysis based on haplotype clusters increased power over single-locus test, and that another association test based on diplotype trend regression analysis outperformed other, more common association approaches^17^,^18^. Therefore, a novel algorithm to combine haplotype cluster- and diplotype-based analysis is suggested.

The people of the very small ethnic minority, Jing^19^, live in compact communities primarily on the three islands of Wanwei, Wutou and Shanxin in the county of Fangchenggang in the province of Guangxi, near the Sino-Vietnamese border. The ancestors of Jing ethnic group had immigrated from the Vietnam since the 15^th^ century. Today, they live in nearby with predominately Han Chinese, the biggest population in China. The Jing people like to eat fish, shrimps, crabs, fish soluble and rice cakes. The daily dishes are mainly fish and shrimps, which are always made into soluble, and it is an inevitable seasoning ingredient at each meal. The typical food of the Jing people is fish soluble, which is also called silurid soluble. It is the traditional seasoning among the Jing people and is made from different kinds of small fishes which are first pickled before made into the soluble. Additionally, the Jing women like to chew betel nuts. Importantly, Jing is a relatively isolated and conservative minority not only in nature environment but also in social network. Furthermore, Jing preserves their custom of intra-ethnic marriage. Consequently, their genetic background may be less heterogeneous within the population (http://www.chinatravel.com/facts/jing-ethnic-minority.htm). Hence, considering that the *CAPN3* and *FRMD5* might play an important role on the etiopathogenesis of CVD, we aimed to investigate the possible association of the integrative variants, haplotypes and diplotypes of the *CAPN3* rs4344713 and *FRMD5* rs524908, as well as several environmental exposures with the lipid homeostasis in the Jing and Han populations.

## Results

### Sociodemographic and clinical characteristics

The sociodemographic and clinical characteristics of the Jing and Han participants are list in Table 1. As both groups were matched by age and gender, there were no statistically significant differences by age and gender. The mean value of body mass index (BMI) in the Jing ethnic minority was significantly higher than that in the Han population. However, the values of systolic blood pressure (SBP) and pulse pressure (PP) in the Jing group were significantly lower than those in the Han group. There were equivalent significant differences between the two ethnic groups for the social lifestyle. The percentage of light-moderate alcohol consumption was significantly higher in the Jing than in the Han individuals, while the % of severe cigarette smoking and severe alcohol consumption was significantly lower in the Jing than in the Han subjects. For serum lipid variables, the levels of total cholesterol (TC), TG and apolipoprotein (Apo) B were significantly higher and those of high-density lipoprotein cholesterol (HDL-C), ApoA1 and the ApoA1/ApoB ratio were significantly lower in the Jing than in the Han participants. However, no difference was noted in fasting serum low-density lipoprotein cholesterol (LDL-C) levels between the two ethnic groups.

### Genotypic and allelic frequencies

**Figure 2:**
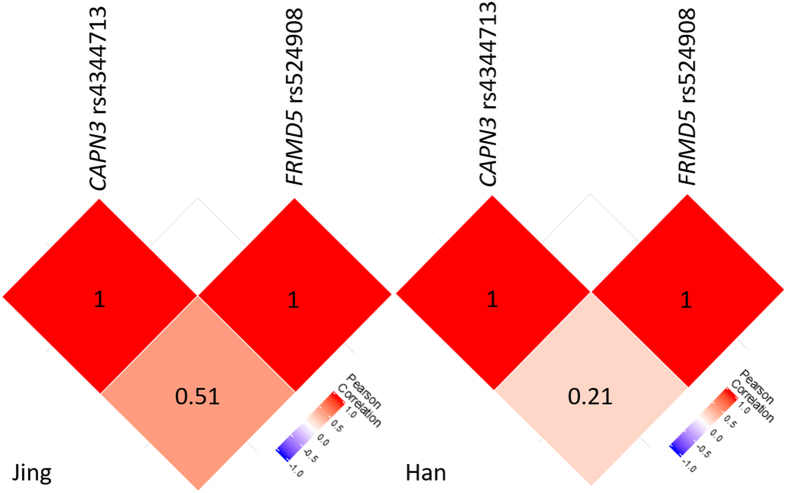
Inter-locus correlation of the *CAPN3* and *FRMD5* in the Jing and Han ethnic groups.

Regarding the analysis of gene frequencies in the *CAPN3* and *FRMD5*, the prevalence of minor allelic, genotypic, haplotypic and diplotypic frequencies of the *CAPN3* rs4344713 and *FRMD5* rs524908 was significantly different between the Jing and Han populations (Table 2). We found that the rare variant or minor allele frequency (MAF) of the *CAPN3* rs4344713 and *FRMD5* rs524908 were significantly higher in the Jing than in the Han subjects (2.93% *vs.* 1.58%, *P* = 0.009 and 21.31% *vs.* 17.78%, *P* = 0.011; respectively). The prevalence of the *CAPN3* rs4344713CC and *FRMD5* rs524908CC genotypes was also significantly higher in the Jing than in the Han ethnic groups (0.25% *vs.* 0.12%, *P* = 0.038 and 5.25% *vs.* 3.90%, *P* = 0.044; respectively). Two variants in the Jing and Han populations were in the Hardy-Weinberg equilibrium (HWE, *P* > 0.05 for all). There was weak linkage disequilibrium (LD) between the two SNPs (*D′* = 0.834, *r*^2^ = 0.075 in Jing, *D′* = 0.761, *r*^2^ = 0.049 in Han, *P* = 0.007). Pearson correlation analysis demonstrated that the *CAPN3* rs4344713 and *FRMD5* rs524908 SNPs were not totally independent from each other (Fig. 2). The frequencies of haplotype and diplotype are list in Table 2. Two haplotypes (rs4344713T-rs524908A and rs4344713T-rs524908C) and two diplotypes (rs4344713TT-rs524908AA and rs4344713TT-rs524908AC) with a frequency > 5%, called “common”, were identified in the Jing and Han populations; respectively. We combined 1 haplotype (rs4344713C-rs524908C both in the Jing and Han) and 3 diplotypes (rs4344713TT-rs524908CC and rs4344713CT-rs524908AC in Han, and rs4344713CT-rs524908CC in Jing) with frequencies between 1% and 5% into one group, called “low-frequency”. The rest of haplotypes and diplotypes with frequencies less than 1% into one group, called “rare”. The haplotype of rs4344713T-rs524908A and diplotype of rs4344713TT-rs524908AA were the commonest, which accounted for over half of the % of each ethnic group.

### Genotypes and serum lipid phenotypes

As shown in Table 3, the levels of LDL-C, ApoB and the ratio of ApoA1 to ApoB in the Jing population were significantly different between the *CAPN3* rs4344713TT and CT/CC or among the *FRMD5* rs524908AA, AC and CC genotypes after the Bonferroni correction (*P ≤ *0.022). There was no association of the two SNPs and serum lipid phenotypes in the Han population.

### Haplotypes and serum lipid phenotypes

After the Bonferroni correction, the levels of TG (rs4344713T-rs524908A and rs4344713C-rs524908A), ApoA1 (rs4344713T-rs524908A, rs4344713T-rs524908C and rs4344713C-rs524908A), ApoB (rs4344713T-rs524908A and rs4344713T-rs524908C) and the ApoA1/ApoB ratio (rs4344713T-rs524908A and rs4344713T-rs524908C) in Jing; and TG (rs4344713C-rs524908A) in Han were significantly different between the haplotype carriers and non-carriers (*P* ≤ 0.019, Table 4). The association analysis based on haplotype cluster increased power over single-locus tests.

### Diplotypes and serum lipid phenotypes

As shown in Table 5, the levels of TG (rs4344713TT-rs524908AA, rs4344713TT-rs524908CC and rs4344713CT/CC-rs524908AA), HDL-C (rs4344713TT-rs524908CC), LDL-C (rs4344713CT/CC-rs524908CC), ApoA1 (rs4344713TT-rs524908AA), ApoB (rs4344713TT-rs524908AA) and ApoA1/ApoB (rs4344713TT-rs524908AA, rs4344713TT-rs524908AC and rs4344713TT-rs524908CC) in Jing; and TG (rs4344713CT/CC-rs524908AA) and ApoA1 (rs4344713TT-rs524908CC) in Han were significantly different between the diplotype carriers and non-carriers after the Bonferroni correction (*P* ≤ 0.023). The association test based on diplotype outperformed other more common single variant association approaches.

### Genotypes, haplotypes and diplotypes and serum lipid phenotypes

Table 6 shows the magnitude and direction of correlation between serum lipid levels and integrative genotypes, haplotypes and diplotypes in the two populations. Many of the examining variants showed significant correlation with serum lipid levels in multiple linear regression analysis; although, these variants did not show significant association with serum lipid levels in the analysis of covariance (ANCOVA). Furthermore, the significantly differences in the LDL-C level change per increment of *CAPN3* genotype was Beta/Se 0.059/0.046 (*P* = 0.016), as well as HDL-C, ApoA1, ApoB levels and the ApoA1/ApoB ratio change per increment of *FRMD5* genotype were −0.359/0.150 (*P* = 0.017); −0.063/0.010 (*P* = 0.009); 0.079/0.010 (*P* = 0.001) and −0.096/0.015 (*P* < 0.001) in the Jing participants, respectively. In addition, the significantly differently in the HDL-C change per increment of their haplotype was Beta/Se 0.535/0.233 (*P* = 0.023), as well as marginal significantly differently in the ApoA1 change per increment of their haplotype was Beta/Se 0.159/0.075 (*P* = 0.033) and the ApoA1/ApoB ratio change per increment of their diplotype was Beta/Se 0.414/0.201 (*P* = 0.040). However, we did not obtain any significant association (having taken correction for multiple comparisons into account) with any of these biomarkers in the Han population of this ethnic-stratified analysis. The novel algorithm to combine haplotype cluster- and diplotype-based analyses is useful for identifying more precise and distinct signals over single-locus tests.

### Relative factors for serum lipid phenotypic parameters

**Figure 3:**
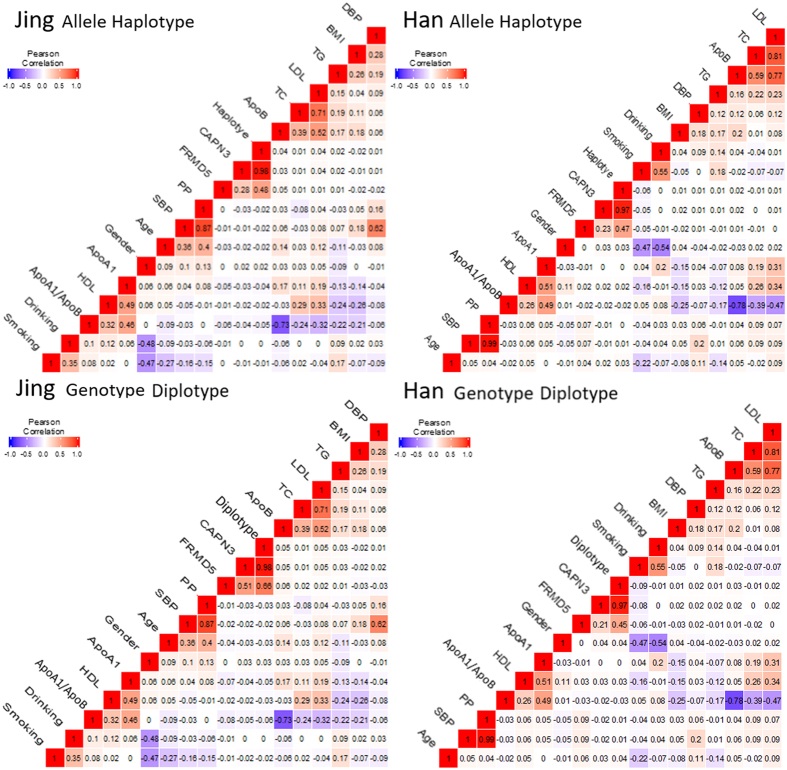
Correlation between environmental exposures and serum lipid variables, as well as the candidate locus.

As shown in Fig. 3, Pearson correlation analysis showed that the integrative variants, haplotype and diplotype evidence connects the *CAPN3* rs4344713 and *FRMD5* rs524908 to lipid variables. Several environmental exposures such as age, gender, cigarette smoking, alcohol consumption and traditional cardiovascular risk factors such as BMI and blood pressure levels were also correlated with serum lipid phenotypic parameters of both ethnic groups.

## Discussion

In the present study, we first confirmed that (*i*) not only the *CAPN3* rs4344713 CT/CC genotype and the *FRMD5* rs524908 CC genotype, but also the rs4344713C-rs524908C haplotype and the rs4344713CT/CC-rs524908CC diplotype were good proxies for higher serum LDL-C and ApoB levels in the Jing but not in the Han populations; (ii) the rs4344713T-rs524908A, rs4344713T-rs524908C and rs4344713C-rs524908A haplotypes were associated with TG and ApoA1 in the Jing particiapnts, as well as associated with TG in the Han subjects; and (iii) the results of our study also suggest a pleiotropic association of the *CAPN3*-*FRMD5* diplotypes with lipid variables in the both Jing and Han populations. In summary, the data presented here suggest differences in lipid variables between the two populations might partially attribute to *CAPN3* rs4344713 and *FRMD5* rs524908 variants, while this heterogeneity needs to be confirmed in other populations in order to better assess its important. Moreover, to combine haplotype cluster- and diplotype-based analyses is useful for identifying more precise and distinct signals over single-locus tests.

A couples of years ago, Schunkert *et al*.^8^ found that the individuals with *CAPN3* rs24212710G allele were positively associated with plasma TG levels (*n* = 153,909, *P *=* *1.7 × 10^−11^) and increased the risk of CVD (*n* = 79,267, *P *=* *5.3 × 10^−3^) in the European population. Likewise, in the same European population’s large-scale association study, the *FRMD5* rs2929282A allele carriers had higher TG levels (*n* = 83,616, *P *=* *2 × 10^−9^) and risk of CVD (*n* = 81,446, *P *=* *2.8 × 10^−3^) than the rs2929282A allele non-carriers. However, no association was seen between the *FRMD5* rs2929282 and BMI (*n* = 122,284, *P *=* *1.1 × 10^−2^). In the present study, we found that the *CAPN3* rs4344713CT/CC genotype and the *FRMD5* rs524908CC genotype were more frequent in the Jing than in the Han populations. The subjects with the *CAPN3* rs4344713CT/CC genotype and the *FRMD5* rs524908CC genotype tend to have higher serum LDL-C and ApoB levels in the Jing ethnic minority. However, no such association was observed in the Han population. The reason for this discrepancy among these studies is not fully understood. It might result from the difference in the genetic background, ethnic LD pattern and/or environmental factors.

On inter-locus interaction analyses, we confirmed the *CAPN3* rs4344713 T > C variant in low LD with the *FRMD5* rs524908 A > C. At least, they are not statistically independent. In single locus analysis, only LDL-C, ApoB levels and the ApoA1/ApoB ratio were associated with the detected variants. However, in haplotype cluster- and diplotype-based analyses, significant associations with pleiotropic lipid variables were found in the *CAPN3-FRMD5* haplotype and diplotype models. These findings indicate that a potential gene-gene interaction might exist between the *CAPN3* rs4344713 and *FRMD5* rs524908 variants. Unfortunately, no previous study has investigated the inter-locus interaction between these variants, and therefore we cannot make comparisons with our results. Although, a statistically significant variant-, haplotype- and diplotype-based association was noted in this study, the biological mechanism underlying these genes and their interactions is still yet to be defined.

When assessing the association of several environmental exposures with lipid variables, we showed that diastolic blood pressure (DBP) and BMI were strongly associated with serum lipid levels in the both Jing and Han populations. A novel extension to the phenome-wide association study (pheWAS) approach^20^, using Bonferroni corrections, permutation testing and estimates of the false discovery rate to consider the strength of results given the number of tests performed demonstrated strongly associated outcomes included DBP and BMI with lipid profiles. They also found novel evidence of effects of BMI on a global self-worth score. Previous studies have reported not only higher DBP^21^ but also both overweight and obesity^22^,^23^ are associated with an increased risk of CVD death and all-cause mortality. Furthermore, achieving and maintaining a healthy weight has a favorable effect on metabolic risk factors (blood pressure, lipids) and low CVD risk. Whatever, we considered these variables as potential confounders and performed a crude statistical analysis and a multivariate analysis adjusting for these variables.

*CAPN3* encodes CAPN3, a heterodimer consisting of a large and a small subunit, is a major intracellular protease^24^, although its function has not been well established. Mutations in this gene^25^ are associated with lipid metabolism. Alternate promoters and alternative splicing result in multiple transcript variants encoding different isoforms and some variants are ubiquitously expressed. Likewise, *FRMD5* encodes FERM-containing protein FRMD5, a p120-catenin interacting protein, regulates lipid metabolic progression^26^. Our study is the first one carried out in a south Chinese population and the first to find a significant association between this integrative variants, haplotype and diplotypes of the *CAPN3* and *FRMD5* with lipid variables. After adjustment for several potential confounders, this association remained significant.

Sampling bias cannot be ignored. In our statistics, the sample is collected in previous specimen library that some members of the intended population are less likely to be included than others. It results in a biased sample, a non-random sample^27^ of a population (non-human or human factors) in which all individuals, or instances, were not equally likely to have been selected^28^. If this is not accounted for, results can be erroneously attributed to the phenomenon under study rather than to the method of sampling. As an ascertainment bias sometimes classified as the inevitable type of bias.

Our analyses benefited from a strong a priori hypothesis, a good sample size for a sub-ethnic study, and a healthy population, making generalizations to other samples less problematic than with clinical samples. However, there are some limitations: first, the association in the present study should be tested in more racially diverse populations. Second, many of our conclusions regarding associations did not survive the FDR correction for multiple testing, so should be considered preliminary. Third, it is not clear why some variants in LD with each other did not show the same genotype-phenotype associations. Moreover, observational analyses may not provide robust evidence of causality, as they are susceptible to confounding, reverse causation and measurement error. The last but not the least, the selected variants may not the accurate and precise hits because tagging SNPs selection bias is inevitable.

In conclusion, integrative variants, haplotypes and diplotypes of the *CAPN3* rs4344713 and *FRMD5* rs524908, as well as DBP and BMI associated with lipid variables in the Jing and Han populations. These associations may contribute to future novel dyslipidemia diagnosis biomarkers and pharmacogenomics therapy targets.

## Materials and Methods

### Ethics statement

Protocols in the present study have approval from the Ethics Committee of the First Affiliated Hospital, Guangxi Medical University (No: Lunshen-2011-KY-Guoji-001; Mar. 7, 2011). Subjects from contributing populations gave written informed consent to participate in epidemiologic investigation, biochemical measurement and genetic analysis. This study was carried out following the rules of the Declaration of Helsinki of 1975 (http://www.wma.net/en/30publications/10policies/b3/), revised in 2008.

### Subjects

The Jing participants in the current study were mainly recruited from three islands: Wanwei, Wutou and Shanxin. Eligibility criteria were (1) ≥ 18 years of age; (2) willing to participate in epidemiologic investigation, biochemical measurement and genetic analysis of this project; (3) their birthplaces and those of their parents, grandparents, and great-grandparents clustered in the abovementioned three islands; (4) their ethnicities and those of their parents, grandparents, and great-grandparents were the same one (Jing); (5) myocardial enzymes, aspartate aminotransferase, alanine aminotransferase, and creatinine results within normal range; and (6) normal electrocardiography (ECG) results. Exclusion criteria were (1) with history of coronary artery disease, stroke, diabetes, hyper- or hypo-thyroids, and chronic hepatic and renal disease; (2) use of lipid lower drugs, including prescription, over-the-counter and nutraceuticals; and (3) other medications known to affect lipid profiles, such as insulin use. After granting informed consent, participants underwent a baseline-screening visit and a fasting blood draw. A total of 1800 Jing subjects were asked to participate in the study and 1674 Jing subjects actually participated^17^. The response rate was 93% in Jing. A total of 52 persons (3.1%) with a history and/or evidence of other diseases were excluded from the Jing samples. The sampling of the Han population was also done in the same place, by the same method. Importantly, the participants of this study were randomly selected from our previous stratified randomized samples, just included 818 Jing subjects and 822 Han participants.

### Biochemical analyses

Protocols for measuring lipid variables have been previously described^29^. All lipid variables were measured in our Clinical Science Experiment Center. The levels of fasting serum TC, TG, HDL-C and LDL-C in the samples were determined by enzymatic methods with commercially available kits. Fasting serum ApoA1 and ApoB levels were assessed by the immuneturbidimetric immunoassay, respectively.

### Tagging SNPs selection

We selected two SNPs in the *CAPN3/FRMD5* with the following steps: (i) CAPN3 gene clusters, which were selected from previous GWAS associated with lipid-metabolism. FRMD5 gene clusters are found to be close to CAPN3 gene clusters and associated with serum lipid level especially TG. (ii) Tagging SNPs, which were established by Haploview (Broad Institute of MIT and Harvard, USA, version 4.2) and functional SNPs predicted to lead to serum lipid changes from current version of online resource (1000 Genome Project Database). (iii) *CAPN3* rs2412710 and *FRMD5* rs2929282, which were selected by the block-based approach. This strategy is enable by the correlations between tagging SNPs as manifested as LD. Although classic is not goal of tagging SNP selection, innovative tagging SNPs selection bias is inevitable.

### Variant genotyping

Genomic DNA was extracted from leucocytes of venous blood using the phenol-chloroform method. Genotyping was performed in each population separately with PCR and Sanger sequencing. The PCR products of the samples were sequenced with a sequencer ABI Prism 3100 Genetic Analyzer (Applied Biosystems, International Equipment Trading Ltd., Vernon Hills, IL, USA) in Shanghai Sangon Biological Engineering Technology & Services Co. Ltd., Shanghai, China^30^.

### Statistical analyses

The statistical analyses were performed with the statistical software SPSS 21.0 (SPSS Inc., Chicago, IL, USA). Quantitative variables were presented as the mean ± SD for those, that are normally distributed, whereas the medians and interquartile ranges for TG, which is not normally distributed. General characteristics between the two groups were compared by the *ANCOVA*. The distributions of the genotype, allele, haplotype and diplotype between the two groups were analyzed by the chi-squared test; The HWE, Pair-wise LD, frequencies of haplotype and diplotype interaction comprising the variants were calculated using Haploview (version 4.2; Broad Institute of MIT and Harvard) and PLINK (version 1.9; Broad Institute of MIT and Harvard) software. The association of the genotypes, haplotypes and diplotypes with lipid variables was tested by the *Univariant*. As 2 loci are not totally independent from each other, we determined these variants represented 1.86 independent loci using Matrix Spectral Decomposition. For all analyses, a Bonferroni test adjusted *P-value* less than 0.025 was considered statistically significant, and the *P-value* between 0.025–0.05 was considered marginal statistically significant. Generalized linear models were used to assess the association of the genotypes (common homozygote genotype = 1, heterozygote genotype = 2, rare homozygote genotype = 3), alleles (the minor allele non-carrier = 1, the minor allele carrier = 2), haplotypes (the haplotype non-carrier = 1, the haplotype carrier = 2) and diplotypes (the diplotype non-carrier = 1, the diplotype carrier = 2) with lipid variables. The model of age, gender, BMI, waist circumference, systolic blood pressure, DBP, pulse pressure, cigarette smoking, alcohol consumption and fasting blood glucose level were adjusted for the statistical analysis. The heart-map of inter-locus models was measured by R software (version 3.3.0).

## Additional Information

**How to cite this article:** Guo, T. *et al*. Integrative variants, haplotypes and diplotypes of the CAPN3 and FRMD5 genes and several environmental exposures associate with serum lipid variables. *Sci. Rep.* **7**, 45119; doi: 10.1038/srep45119 (2017).

**Publisher's note:** Springer Nature remains neutral with regard to jurisdictional claims in published maps and institutional affiliations.

## Figures and Tables

**Figure 1:**
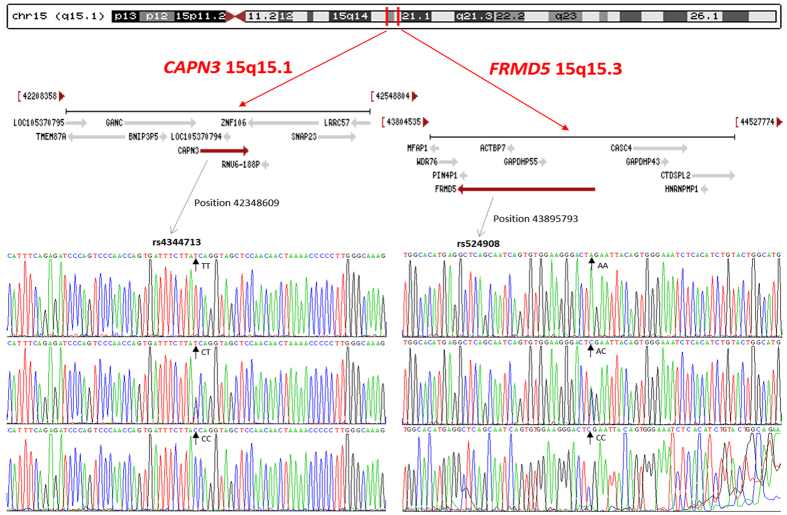
The positions of the *CAPN3* and *FRMD5* variants.

**Table 1:** Sociodemographic and clinical characteristics of the Jing and Han populations.

Characteristics	Jing(n = 819)	Han(n = 821)	*t(X*^*2*^)	*P-value*
Age (years)^1^	56.97 ± 12.70	57.27 ± 12.86	1.434	0.152
Gender(Male/Female)	411/408	413/408	0.002	0.961
Body mass index (kg/m^2^)	23.42 ± 3.18	22.82 ± 3.19	5.431	< 0.001
Systolic blood pressure (mmHg)	132.02 ± 21.66	137.35 ± 22.35	−2.861	0.004
Diastolic blood pressure (mmHg)	80.78 ± 10.64	81.44 ± 10.40	−1.794	0.073
Pulse pressure (mmHg)	51.24 ± 17.27	55.91 ± 21.00	−2.586	0.010
Cigarette smoking [n (%)]				
Nonsmoker	656 (80.1)	619 (75.4)		
≤20 Cigarette smoking/day	38 (4.6)	38 (4.6)		
>20 Cigarette smoking/day	125 (15.3)	164 (20.0)	6.334	0.042
Alcohol consumption [n (%)]				
Nondrinker	627 (76.6)	558 (68.0)		
≤25 g/day	95 (11.6)	60 (7.3)		
>25 g/day	97 (11.8)	203 (24.7)	49.372	< 0.001
Total cholesterol (mmol/L)	5.29 ± 0.89	4.99 ± 0.87	9.772	< 0.001
Triglyceride (mmol/L)^2^	1.51 (1.18)	1.37 (1.09)	−6.317	< 0.001
HDL-C (mmol/L)	1.77 ± 0.54	1.80 ± 0.46	−2.149	0.032
LDL-C (mmol/L)	2.87 ± 0.43	2.85 ± 0.43	1.269	0.204
Apolipoprotein (Apo) A1 (g/L)	1.31 ± 0.25	1.33 ± 0.20	−2.988	0.003
ApoB (g/L)	1.07 ± 0.25	1.05 ± 0.24	2.576	0.010
ApoA1/ApoB	1.29 ± 0.38	1.34 ± 0.38	−3.769	< 0.001

*HDL-C*, high-density lipoprotein cholesterol; *LDL-C*, low-density lipoprotein cholesterol. ^1^Mean ± SD determined by *t*-test. ^2^Median (interquartile range) tested by the *Wilcoxon-Mann-Whitney test*.

**Table 2:** Distribution of variants, haplotypes and diplotypes in the Jing and Han populations[n (%)].

Type		Jing (n = 819)	Han (n = 821)	*X*^*2*^	*P*-value
Allele	*CAPN3* rs4344713-MAF	48 (2.93)	26 (1.58)	6.747	0.009
	*FRMD5* rs524908-MAF	349 (21.31)	292 (17.78)	6.474	0.011
Genotype	*CAPN3* rs4344713-TT	773 (94.38)	796 (96.95)		
	*CAPN3* rs4344713-CT	44 (5.37)	24 (2.93)		
	*CAPN3* rs4344713-CC	2 (0.25)	1 (0.12)	6.550	0.038
	HWE(*P-value*)	0.111	0.075		
	*FRMD5* rs524908-AA	513 (62.64)	561 (68.33)		
	*FRMD5* rs524908-AC	263 (32.11)	228 (27.77)		
	*FRMD5* rs524908-CC	43 (5.25)	32 (3.90)	6.251	0.044
	HWE(*P-value*)	0.225	0.150		
Haplotype	*CAPN3- FRMD5* T-A	1283 (78.33)	1343 (81.55)		
	*CAPN3- FRMD5* T-C	307 (18.74)	273 (16.87)		
	*CAPN3- FRMD5* C-A	6 (0.37)	7 (0.67)		
	*CAPN3- FRMD5* C-C	42 (2.56)	19 (0.91)	11.975	0.001
Diplotype	*CAPN3- FRMD5* TT-AA	509 (62.15)	556 (67.72)		
	*CAPN3- FRMD5* TT-AC	261 (31.87)	215 (26.19)		
	*CAPN3- FRMD5* TT-CC	3 (0.37)	25 (3.05)		
	*CAPN3- FRMD5* CT-AA	2 (0.24)	4 (0.49)		
	*CAPN3- FRMD5* CT-AC	2 (0.24)	13 (1.58)		
	*CAPN3- FRMD5* CT-CC	40 (4.89)	7 (0.85)		
	*CAPN3- FRMD5* CC-AA	2 (0.24)	1 (0.12)		
	*CAPN3- FRMD5* CC-AC	0 (0)	0 (0)		
	*CAPN3- FRMD5* CC-CC	0 (0)	0 (0)	56.040	< 0.001

*MAF*, minor allele frequency; *HWE*, Hardy-Weinberg equilibrium; Haplotypes and diplotypes were in the order of *CAPN3* rs4344713 and *FRMD5* rs524908.

**Table 3:** Associations of the *CAPN3* and *FRMD5* variants and serum lipid phenotypes in the Jing and Han populations.

Allele/Genotype	TC (mmol/L)^1^	TG (mmol/L)^2^	HDL-C (mmol/L)	LDL-C (mmol/L)	ApoA1 (g/L)	ApoB (g/L)	ApoA1/ApoB
***CAPN3*** **rs4344713 T > C**							
Jing							
TT	5.29 ± 0.90	1.50 (1.17)	1.81 ± 0.46	2.84 ± 0.43	1.31 ± 0.25	1.07 ± 0.25	1.29 ± 0.38
CT/CC	5.34 ± 0.78	1.59 (1.28)	1.77 ± 0.42	2.95 ± 0.42	1.27 ± 0.23	1.11 ± 0.26	1.21 ± 0.42
*F*	0.222	−1.517	2.564	5.763	3.021	5.245	6.510
*P*	0.637	0.129	0.110	0.016	0.082	0.022	0.011
Han							
TT	4.98 ± 0.79	1.36 (1.09)	1.86 ± 0.47	2.86 ± 0.43	1.33 ± 0.20	1.04 ± 0.24	1.34 ± 0.38
CT/CC	4.99 ± 0.88	1.50 (1.17)	1.76 ± 0.54	2.90 ± 0.43	1.33 ± 0.19	1.07 ± 0.24	1.29 ± 0.32
*F*	0.007	−1.918	0.429	0.403	0.030	0.542	0.861
*P*	0.935	0.055	0.513	0.526	0.862	0.462	0.354
***FRMD5* rs524908 A > C**							
Jing							
AA	5.28 ± 0.91	1.50 (1.17)	1.81 ± 0.48	2.82 ± 0.46	1.32 ± 0.26	1.06 ± 0.25	1.31 ± 0.38
AC	5.30 ± 0.87	1.54 (1.24)	1.80 ± 0.44	2.85 ± 0.42	1.28 ± 0.21	1.08 ± 0.25	1.25 ± 0.37
CC	5.36 ± 0.79	1.59 (1.28)	1.77 ± 0.41	2.97 ± 0.41	1.28 ± 0.23	1.13 ± 0.26	1.21 ± 0.43
*F*	0.371	−1.679	1.180	4.584	3.674	5.847	7.701
*P*	0.690	0.093	0.307	0.010	0.026	0.003	0.000
Han							
AA	4.87 ± 0.80	1.36 (1.07)	1.81 ± 0.51	2.86 ± 0.42	1.33 ± 0.21	1.04 ± 0.23	1.34 ± 0.39
AC	4.98 ± 0.84	1.46 (1.12)	1.75 ± 0.57	2.87 ± 0.43	1.33 ± 0.20	1.05 ± 0.24	1.34 ± 0.36
CC	4.99 ± 0.89	1.49 (1.13)	1.70 ± 0.39	2.89 ± 0.44	1.27 ± 0.18	1.09 ± 0.21	1.21 ± 0.31
*F*	1.400	−1.525	0.598	0.082	2.282	0.294	2.157
*P*	0.247	0.127	0.550	0.922	0.102	0.746	0.116

*TC*, total cholesterol; *TG*, triglyceride; *HDL-C*, high-density lipoprotein cholesterol; *LDL-C*, low-density lipoprotein cholesterol; *ApoA1*, apolipoprotein A1; *ApoB*, apolipoprotein B; *ApoA1/ApoB*, the ratio of apolipoprotein A1 to apolipoprotein B. ^1^Mean ± SD determined by the *ANCOVA*. ^2^Median (interquartile range) tested by the *Wilcoxon-Mann-Whitney test* or *Kruskal-Wallis test*. A *P-*value less than 0.025 was considered statistically significant after the Bonferroni correction.

**Table 4:** Associations of the haplotypes and serum lipid phenotypes in the Jing and Han populations.

Haplotype	Group	TC (mmol/L)^1^	TG (mmol/L)^2^	HDL-C (mmol/L)	LDL-C (mmol/L)	ApoA1 (g/L)	ApoB (g/L)	ApoA1/ApoB
T-A	Jing							
	Carrier	5.28 ± 0.91	1.50 (1.17)	1.81 ± 0.47	2.85 ± 0.43	1.31 ± 0.25	1.06 ± 0.25	1.30 ± 0.38
	Non-carrier	5.31 ± 0.84	1.55 (1.24)	1.79 ± 0.43	2.86 ± 0.45	1.28 ± 0.22	1.09 ± 0.25	1.24 ± 0.38
	*F*	0.558	−2.652	2.147	0.735	9.374	11.692	18.321
	*P*	0.455	0.008	0.143	0.391	0.002	0.001	0.000
	Han							
	Carrier	4.99 ± 0.88	1.36 (1.08)	1.76 ± 0.56	2.87 ± 0.43	1.33 ± 0.20	1.04 ± 0.24	1.34 ± 0.38
	Non-carrier	4.96 ± 0.83	1.46 (1.12)	1.79 ± 0.49	2.87 ± 0.42	1.32 ± 0.20	1.05 ± 0.23	1.32 ± 0.35
	*F*	1.205	−2.212	0.007	0.009	0.779	0.776	2.992
	*P*	0.272	0.027	0.936	0.926	0.378	0.378	0.084
T-C	Jing							
	Carrier	5.31 ± 0.85	1.54 (1.24)	1.79 ± 0.43	2.85 ± 0.46	1.28 ± 0.22	1.09 ± 0.25	1.24 ± 0.38
	Non-carrier	5.29 ± 0.90	1.51 (1.17)	1.81 ± 0.47	2.85 ± 0.43	1.31 ± 0.25	1.06 ± 0.25	1.30 ± 0.38
	*F*	0.399	−2.016	0.644	0.012	5.783	6.880	11.252
	*P*	0.528	0.044	0.422	0.913	0.016	0.009	0.001
	Han							
	Carrier	4.96 ± 0.84	1.46 (1.12)	1.78 ± 0.49	2.87 ± 0.42	1.32 ± 0.20	1.05 ± 0.23	1.32 ± 0.36
	Non-carrier	4.99 ± 0.88	1.37 (1.09)	1.76 ± 0.55	2.87 ± 0.43	1.33 ± 0.20	1.04 ± 0.24	1.34 ± 0.38
	*F*	1.321	−1.520	0.020	0.023	0.775	0.383	2.109
	*P*	0.250	0.128	0.888	0.881	0.379	0.536	0.147
C-A	Jing							
	Carrier	5.36 ± 0.58	2.01 (1.77)	1.44 ± 0.38	2.97 ± 0.28	1.13 ± 0.16	1.16 ± 0.20	0.99 ± 0.18
	Non-carrier	5.29 ± 0.89	1.51 (1.17)	1.80 ± 0.46	2.85 ± 0.44	1.31 ± 0.25	1.07 ± 0.25	1.29 ± 0.38
	*F*	0.057	−3.262	3.648	0.752	5.531	0.463	4.548
	*P*	0.811	0.001	0.056	0.386	0.019	0.496	0.033
	Han							
	Carrier	5.23 ± 0.70	2.02 (1.77)	1.76 ± 0.51	3.04 ± 0.31	1.34 ± 0.26	1.21 ± 0.23	1.15 ± 0.42
	Non-carrier	4.99 ± 0.87	1.37 (1.09)	1.77 ± 0.54	2.87 ± 0.43	1.33 ± 0.20	1.05 ± 0.24	1.34 ± 0.38
	*F*	0.102	−3.179	0.007	0.625	0.526	3.846	1.130
	*P*	0.749	0.001	0.932	0.429	0.468	0.050	0.288
C-C	Jing							
	Carrier	5.32 ± 0.80	1.55 (1.22)	1.80 ± 0.40	2.95 ± 0.43	1.28 ± 0.23	1.11 ± 0.26	1.23 ± 0.43
	Non-carrier	5.29 ± 0.89	1.51 (1.18)	1.80 ± 0.46	2.85 ± 0.43	1.31 ± 0.25	1.07 ± 0.25	1.29 ± 0.38
	*F*	0.090	−0.691	1.265	4.892	1.327	4.828	4.282
	*P*	0.764	0.490	0.261	0.027	0.249	0.028	0.039
	Han							
	Carrier	4.93 ± 0.78	1.48 (1.16)	1.87 ± 0.46	2.88 ± 0.44	1.33 ± 0.18	1.05 ± 0.24	1.31 ± 0.29
	Non-carrier	4.99 ± 0.87	1.37 (1.09)	1.76 ± 0.54	2.87 ± 0.43	1.33 ± 0.20	1.05 ± 0.24	1.34 ± 0.38
	*F*	0.015	−0.958	0.505	0.151	0.191	0.001	0.332
	*P*	0.901	0.338	0.477	0.698	0.662	0.976	0.564

*TC*, total cholesterol; *TG*, triglyceride; *HDL-C*, high-density lipoprotein cholesterol; *LDL-C*, low-density lipoprotein cholesterol; *ApoA1*, apolipoprotein A1; *ApoB*, apolipoprotein B; *ApoA1/ApoB*, the ratio of apolipoprotein A1 to apolipoprotein B. ^1^Mean ± SD determined by the *ANCOVA*. ^2^Median (interquartile range) tested by the *Wilcoxon-Mann-Whitney test*. A *P-*value less than 0.025 was considered statistically significant after the Bonferroni correction.

**Table 5:** Associations of the diplotypes and serum lipid phenotypes in the Jing and Han populations.

Diplotype	Group	TC (mmol/L)^1^	TG (mmol/L)^2^	HDL-C (mmol/L)	LDL-C (mmol/L)	ApoA1 (g/L)	ApoB (g/L)	ApoA1/ApoB
TT-AA	Jing							
	Carrier	5.28 ± 0.92	1.50 (1.17)	1.81 ± 0.48	2.85 ± 0.42	1.32 ± 0.26	1.06 ± 0.25	1.31 ± 0.38
	Non-carrier	5.31 ± 0.85	1.55 (1.24)	1.79 ± 0.43	2.85 ± 0.46	1.28 ± 0.22	1.09 ± 0.25	1.24 ± 0.38
	*F*	0.315	−2.342	0.954	0.063	8.591	7.766	14.934
	*P*	0.575	0.019	0.329	0.801	0.003	0.005	0.000
	Han							
	Carrier	4.99 ± 0.89	1.36 (1.07)	1.75 ± 0.57	2.86 ± 0.43	1.33 ± 0.20	1.04 ± 0.24	1.34 ± 0.39
	Non-carrier	4.97 ± 0.84	1.46 (1.12)	1.80 ± 0.50	2.87 ± 0.42	1.33 ± 0.20	1.05 ± 0.23	1.33 ± 0.36
	*F*	0.339	−2.130	0.214	0.062	0.022	0.468	1.332
	*P*	0.560	0.033	0.644	0.803	0.882	0.494	0.249
TT-AC	Jing							
	Carrier	5.30 ± 0.86	1.54 (1.23)	1.80 ± 0.44	2.82 ± 0.46	1.28 ± 0.22	1.08 ± 0.25	1.25 ± 0.37
	Non-carrier	5.29 ± 0.91	1.51 (1.17)	1.81 ± 0.47	2.86 ± 0.42	1.32 ± 0.26	1.06 ± 0.25	1.30 ± 0.39
	*F*	0.042	−1.257	0.014	2.643	4.011	2.115	5.903
	*P*	0.837	0.209	0.906	0.104	0.045	0.146	0.015
	Han							
	Carrier	4.98 ± 0.85	1.46 (1.10)	1.81 ± 0.51	2.86 ± 0.42	1.34 ± 0.20	1.04 ± 0.23	1.35 ± 0.36
	Non-carrier	4.99 ± 0.88	1.37 (1.09)	1.75 ± 0.56	2.87 ± 0.43	1.32 ± 0.20	1.05 ± 0.24	1.33 ± 0.38
	*F*	0.025	−0.738	0.362	0.003	0.758	0.011	0.000
	*P*	0.873	0.461	0.547	0.959	0.384	0.917	0.984
TT-CC	Jing							
	Carrier	5.55 ± 0.93	2.00 (1.84)	1.41 ± 0.24	3.05 ± 0.39	1.20 ± 0.11	1.28 ± 0.14	0.95 ± 0.16
	Non-carrier	5.29 ± 0.89	1.51 (1.17)	1.81 ± 0.46	2.85 ± 0.43	1.31 ± 0.25	1.07 ± 0.25	1.29 ± 0.38
	*F*	0.978	−2.935	5.460	1.522	1.697	4.631	5.013
	*P*	0.323	0.003	0.020	0.218	0.193	0.032	0.025
	Han							
	Carrier	4.92 ± 0.82	1.49 (1.19)	1.68 ± 0.41	2.90 ± 0.45	1.26 ± 0.18	1.09 ± 0.21	1.20 ± 0.32
	Non-carrier	4.99 ± 0.87	1.37 (1.09)	1.77 ± 0.55	2.87 ± 0.43	1.33 ± 0.20	1.04 ± 0.24	1.34 ± 0.38
	*F*	1.419	−1.756	0.850	0.018	5.181	0.554	4.185
	*P*	0.234	0.079	0.357	0.893	0.023	0.457	0.041
CT/CC-AA	Jing							
	Carrier	5.47 ± 0.61	2.01 (1.70)	1.47 ± 0.44	2.99 ± 0.31	1.14 ± 0.17	1.16 ± 0.21	1.01 ± 0.20
	Non-carrier	5.29 ± 0.89	1.51 (1.17)	1.81 ± 0.46	2.85 ± 0.44	1.31 ± 0.25	1.07 ± 0.25	1.29 ± 0.38
	*F*	0.316	−2.625	2.562	0.687	3.774	0.284	2.952
	*P*	0.574	0.009	0.110	0.407	0.052	0.594	0.086
	Han							
	Carrier	5.26 ± 0.73	1.97 (1.71)	1.80 ± 0.52	3.05 ± 0.33	1.36 ± 0.27	1.20 ± 0.23	1.18 ± 0.43
	Non-carrier	4.98 ± 0.87	1.36 (1.09)	1.76 ± 0.54	2.87 ± 0.43	1.33 ± 0.20	1.04 ± 0.24	1.34 ± 0.38
	*F*	0.183	−2.938	0.005	0.704	0.701	3.237	0.822
	*P*	0.669	0.003	0.942	0.401	0.403	0.072	0.365
CT/CC-AC	Jing							
	Carrier	5.00 ± 1.16	1.63 (1.56)	1.60 ± 0.49	2.68 ± 0.62	1.24 ± 0.19	1.10 ± 0.21	1.15 ± 0.22
	Non-carrier	5.29 ± 0.89	1.51 (1.18)	1.80 ± 0.46	2.85 ± 0.43	1.31 ± 0.25	1.07 ± 0.25	1.29 ± 0.38
	*F*	0.992	−0.678	2.302	0.547	0.571	0.047	0.793
	*P*	0.319	0.497	0.129	0.460	0.450	0.829	0.373
	Han							
	Carrier	5.00 ± 0.80	1.49 (1.20)	1.87 ± 0.49	2.89 ± 0.46	1.32 ± 0.19	1.05 ± 0.25	1.32 ± 0.31
	Non-carrier	4.99 ± 0.87	1.37 (1.09)	1.76 ± 0.54	2.87 ± 0.43	1.33 ± 0.20	1.05 ± 0.24	1.34 ± 0.38
	*F*	0.319	−1.747	0.457	0.375	0.395	0.000	0.288
	*P*	0.572	0.081	0.499	0.540	0.530	0.983	0.592
CT/CC-CC	Jing							
	Carrier	5.34 ± 0.78	1.51 (1.17)	1.81 ± 0.40	2.96 ± 0.42	1.29 ± 0.23	1.11 ± 0.27	1.24 ± 0.44
	Non-carrier	5.29 ± 0.90	1.52 (1.18)	1.80 ± 0.46	2.84 ± 0.44	1.31 ± 0.25	1.07 ± 0.25	1.29 ± 0.38
	*F*	0.295	−0.558	0.662	6.095	1.038	4.974	3.763
	*P*	0.587	0.577	0.416	0.014	0.308	0.026	0.053
	Han							
	Carrier	4.52 ± 0.61	1.04 (0.93)	1.85 ± 0.23	2.81 ± 0.36	1.36 ± 0.16	1.08 ± 0.20	1.30 ± 0.24
	Non-carrier	4.99 ± 0.87	1.37 (1.10)	1.76 ± 0.54	2.87 ± 0.43	1.33 ± 0.20	1.05 ± 0.24	1.34 ± 0.38
	*F*	2.334	−1.955	0.018	0.342	0.007	0.015	0.094
	*P*	0.127	0.051	0.893	0.559	0.933	0.903	0.760

*TC*, total cholesterol; *TG*, triglyceride; *HDL-C*, high-density lipoprotein cholesterol; *LDL-C*, low-density lipoprotein cholesterol; *ApoA1*, apolipoprotein A1; *ApoB*, apolipoprotein B; *ApoA1/ApoB*, the ratio of apolipoprotein A1 to apolipoprotein B. ^1^Mean ± SD determined by the *ANCOVA*. ^2^Median (interquartile range) tested by the *Wilcoxon-Mann-Whitney test*. A *P-*value less than 0.025 was considered statistically significant after the Bonferroni correction.

**Table 6:** Association of the variants, haplotypes and diplotypes of the *CAPN3* and *FRMD5* and serum lipid variables.

Lipid variable	Gene	Type	Beta	Std.error	Chi-square	*P*-value
**Jing**						
HDL-C	*FRMD5*	Genotype	−0.359	0.150	5.727	0.017
HDL-C	*CAPN3-FRMD5*	Diplotype	0.535	0.235	5.191	0.023
LDL-C	*CAPN3*	Genotype	0.059	0.046	2.410	0.016
ApoA1	*FRMD5*	Genotype	−0.063	0.010	−2.628	0.009
ApoA1	*CAPN3-FRMD5*	Haplotype	0.159	0.075	4.555	0.033
ApoB	*FRMD5*	Genotype	0.079	0.010	3.301	0.001
ApoA1/ApoB	*FRMD5*	Genotype	−0.096	0.015	−4.070	0.000
ApoA1/ApoB	*CAPN3-FRMD5*	Diplotype	0.414	0.201	4.236	0.040

There was no significantly association in the Han population.
